# Terpolymerization of Ethylene with Hexene and Styrene Derivatives by Half-Sandwich Scandium Catalyst

**DOI:** 10.3390/polym16162290

**Published:** 2024-08-14

**Authors:** Xiaochun Mu, Xuefei Leng, Chuanchuan Liu, Qiang Yao, Yang Li

**Affiliations:** 1 Key Laboratory of Bio-Based Polymeric Materials Technology and Application of Zhejiang Province, Ningbo Institute of Materials Technology and Engineering, Chinese Academy of Sciences, Ningbo 315201, China; muxcdlut@163.com (X.M.); yaoqiang@nimte.ac.cn (Q.Y.); 2 SINOPEC Ningbo New Materials Research Institute Company Limited, Ningbo 315201, China; liuchch697.zhlh@sinopec.com; 3 State Key Laboratory of Fine Chemicals, Department of Polymer Science and Engineering, School of Chemical Engineering, Dalian University of Technology, Dalian 116024, China

**Keywords:** functional polyethylene, terpolymerization, hexene, styrene derivatives

## Abstract

The terpolymerization of ethylene with hexene and styrene derivatives was achieved with a rare earth metal catalyst (C_5_Me_4_SiMe_3_)Sc(CH_2_C_6_H_4_NMe_2_-*o*)_2_ to prepare functional polyethylene. The catalyst system exhibited high activity in the terpolymerization of ethylene with hexene and amine-substituted styrene, affording terpolymers a moderate molecular weight and a unimodal molecular weight distribution. In addition, the comonomer content of the terpolymers can be controlled by changing the feeding ratio of monomers. The aliphatic region of the ^13^C NMR spectra reveals that the structural units of the comonomers are separately incorporated into the polyethylene backbone. Terpolymers containing styrene derivatives exhibit enhanced tensile strength and significantly improve hydrophilic properties.

## 1. Introduction

Since the development of Ziegler–Natta and Phillips catalysts in the 1950s, polyethylene (PE) has become one of the most important commercial synthetic polymers and plays a critical role in modern society. However, the nonpolar characteristic of polyethylene severely limits its applications in various fields such as printing inks, paints and adhesives [1,2,3]. Introducing some polar functional groups into the polyethylene backbone is an effective method to improve its properties such as adhesion, compatibility and dyeability [4,5,6,7]. Polymer post-functionalization is the most commonly used method to introduce functional groups into polyethylene in industry. Nevertheless, this process needs to be carried out under harsh conditions, and is usually accompanied with undesirable and uncontrollable side reactions, such as chain scission or cross-linking [8,9].

The transition-metal-catalyzed copolymerization of non-polar olefins, such as ethylene, with heteroatom-functionalized polar olefins is a method for synthesizing functionalized polyolefins. This approach is more efficient and economical than post-functionalization, so it has received extensive attention from both academia and industry. Many early transition-metal and late transition-metal catalysts have been reported for the copolymerization of ethylene with polar vinyl monomers or long-chain polar olefinic monomers [10,11,12,13,14,15,16,17,18,19].

Compared to copolymerization, terpolymerization can regulate polymer properties more precisely and widely, significantly expanding the range of its application [20]. Wu realized highly efficient terpolymerization of ethylene and propylene with 5-ethylidene-2-norbornene (ENB) via a vanadium complexes system, affording ethylene-propylene-ENB terpolymers (EPDMs) with high ENB unit content and an ultra-high molecular weight [21]. Liu succeeded in high-temperature solution polymerization of ethylene, 1-octene and 5-vinyl-2-norbornene by using a metallocene catalyst. The resulting terpolymer was further post-treated with thiol-ene click chemistry and reactive extrusion with bis-dioxaborolane to obtain a dynamically cross-linked polyolefin elastomer [22]. Li achieved terpolymerization of ethylene, 1-tetradecene and 9-(but-3-en-1-yl)anthracene using a metallocene catalyst. The polyolefin elastomers obtained by post-modification of the terpolymer with dioxaborolane maleimide exhibit a high elastic recovery rate and good mechanical properties [23]. Nomura used aryloxo-modified metallocene catalysts to perform terpolymerization of ethylene with styrene and α-olefins (1-hexene, 1-octene) or 1, 7-octendiene, affording terpolymers with adjustable component contents [24,25].

**Scheme 1:**
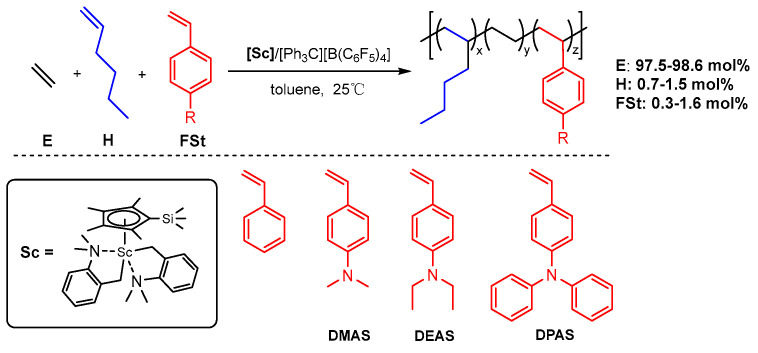
Terpolymerization of ethylene with hexene and styrene or styrene derivatives.

Although there have been some studies on the synthesis of polyolefins by terpolymerization, the preparation of terpolymers containing functionalized monomers has not been reported. The rare earth metal complexes have shown excellent performance as coordination polymerization catalysts in the synthesis of functionalized polyolefins [26,27,28]. Particularly in the copolymerization of olefins with styrene or styrene derivatives, rare earth metal catalysts have shown high activity, which is difficult to achieve with early or late transition metals [29,30,31,32,33]. This paper reports the terpolymerization of ethylene with hexene and styrene derivatives using a half-sandwich scandium alkyl catalyst to prepare functional polyethylenes (Scheme 1). The catalyst system shows high activity for the terpolymerization, and terpolymers with adjustable comonomer content and a moderate molecular weight are obtained. This study provides a promising method for the synthesis of polyethylene with enhanced mechanical and surface properties.

## 2. Materials and Methods

### 2.1. General Methods and Materials

All manipulations of air- and moisture-sensitive compounds were performed either under a dry and oxygen-free nitrogen atmosphere by using standard Schlenk techniques or in a Vigor glovebox. All solvents were purified via a SPS-800 solvent purification system (Mbraun, Stratham, NH, USA) and stored over fresh Na chips in the glovebox. Hexene and styrene were dried by stirring with CaH_2_ overnight, vacuum-distilled and degassed by three freeze–pump–thaw cycles. Styrene derivatives, including *p*-N, N-dimethylaminostyrene (DMAS), *p*-N, N-diethylaminostyrene (DEAS) and *p*-N, N-diphenylaminostyrene (DPAS), were synthesized according to the literature [34]. (C_5_Me_4_SiMe_3_)Sc(CH_2_C_6_H_4_NMe_2_-*o*)_2_ and [Ph_3_C][B(C_6_F_5_)_4_] were prepared according to the literature [35,36].

### 2.2. Characterization

^1^H NMR and ^13^C NMR spectra of the obtained terpolymers were recorded on a JEOL-ECZ600R spectrometer in 1,1,2,2-C_2_D_2_Cl_4_ at 120 °C. The molecular weights and molecular weight distributions of the terpolymers were measured by gel permeation chromatography (GPC) on a Polymer Char apparatus using 1,2,4-trichlorobenzene as an eluent at a flow rate of 1.0 mL/min at 160 °C, with narrow-molecular-weight polystyrene samples as standards for calculations. Differential scanning calorimetry (DSC) was performed with an indium-calibrated DSC1 instrument (Mettler Toledo Corp., Zürich, Switzerland). The samples were sealed in aluminum pans, and an empty pan was sealed and used as a reference. The sealed pans were scanned at a heating and cooling rate of 10 °C/min under nitrogen atmosphere. The melting temperature was taken at the peak. The crystallinity (*X*_c_) was calculated by comparison with the heat of fusion of a perfectly crystalline polyethylene, i.e., 293 J/g [37]. All tensile properties tests were carried out according to the ASTM standard using a Universal Test Machine (INSTRON 5965, Boston, MA, USA) at 25 °C, and the data reported were averaged over five experiments. The water contact angle was measured by an OCA-20 contact angle detector at room temperature.

### 2.3. Typical Terpolymerization Procedure

As a typical reaction, terpolymerization was carried out in a 2 L autoclave reactor equipped with a mechanical stirring blade. Before conducting the terpolymerization, the reactor was vacuumed at 110 °C for drying, followed by three purges with nitrogen and ethylene in turn. The toluene solution containing hexene (20–40 mmol) and styrene or styrene derivatives (10–30 mmol) was injected into the reactor at room temperature, and ethylene (0.5 MPa) was introduced into the reactor. Ethylene was equilibrated by stirring at the desired reaction temperature for 10 min with a stirring speed of 200 rpm. A toluene solution (30 mL) of (C_5_Me_4_SiMe_3_)Sc(CH_2_C_6_H_4_NMe_2_-*o*)_2_ (20.2 mg, 40 μmol) and 1 equivalent [Ph_3_C][B(C_6_F_5_)_4_] (36.9 mg, 40 μmol) was injected into the reactor to initiate terpolymerization. The terpolymerization was conducted at 25 °C under a constant ethylene pressure of 0.5 MPa for 15 min. The polymerization was terminated with the addition of methanol, then the resulting mixture was poured into a large amount of methanol to precipitate the polymer product. The precipitated polymer was filtered and dried under vacuum at 60 °C to a constant weight.

## 3. Results

### 3.1. Terpolymerization of Ethylene with Hexene and Styrene

First, the terpolymerization of ethylene with hexene and styrene was explored under different feeding ratios of hexene and styrene. The catalyst system showed high activity for the terpolymerization and the activity increased upon increasing the styrene initial concentration under similar ethylene and hexene concentration conditions (Table 1, runs 1–3). All poly(ethylene-hexene-styrene) samples were examined via ^1^H NMR to reveal their compositions (Figure S1). As the styrene feeding amount increased, the incorporation amount of styrene in the terpolymers increased linearly while the incorporation of hexene decreased slightly. The ratios of [St]/[Hex] and [St]_0_/[Hex]_0_ were basically constant, indicating that styrene mainly replaces ethylene rather than hexene during the polymerization ([St] and [Hex] refer to the content of styrene and hexene in the terpolymer, [St]_0_ and [Hex]_0_ refer to the feeding amounts of styrene and hexene, respectively). The conversion of comonomers is shown in Table S1. The conversion of hexene and styrene decreased when the feeding amount of styrene increased, and the conversion of styrene was higher than that of hexene when the two comonomers were in the same feeding amount (Table S1, run 2), indicating that the coordination insertion of styrene was easier than that of hexene. When the relative feeding ratio of hexene to styrene was increased (Table 1, runs 3–5), the polymerization activity improved, and the incorporation amount of hexene in the terpolymers increased, while the incorporation amount of styrene decreased. The ratios of [St]/[Hex] and [St]_0_/[Hex]_0_ gradually decreased, indicating that the increase in the amount of hexene fed was much greater than its consumption rate, so its conversion rate decreased.

Ultimately, terpolymers with a moderate molecular weight (9.7–12.9 × 10^4^ Da) and a unimodal molecular weight distribution (PDI = 1.7–2.4) were obtained. The molecular weight decreased slightly with the increase in the styrene feeding amount or the relative feeding amounts of hexene to styrene, which may be due to the β-hydrogen elimination reaction during the polymerization process [38]. The copolymerization of hexene and styrene was also attempted, but only syndiotactic polystyrene was obtained, possibly due to the significant difference in polymerization activity between the two monomers [35,39]. DSC analysis (Figure S3) revealed that the melting temperature (*T*_m_) and the crystallinity (*X*_c_) of the terpolymers decreased as the incorporation amount of comonomers increased, indicating that the resultant polymers possessed uniform compositions and comonomers are distributed along the polyethylene backbone.

**Figure 1:**
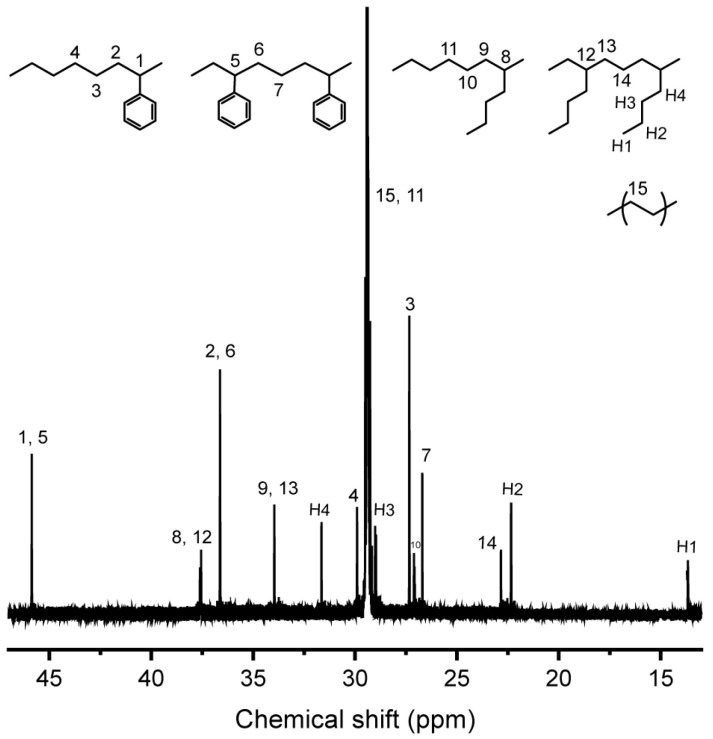
Aliphatic region of ^13^C NMR spectra of poly(ethylene-hexene-styrene).

The representative ^13^C NMR spectrum of poly(ethylene-co-hexene-co-styrene) (Table 1, run 3) is presented in Figure 1. According to the literature, the aliphatic region of the ^13^C NMR spectra of the terpolymer provides some information about the sequence distribution of this new type of product [20,24]. Resonances observed at 45.9 ppm and 36.6 ppm are attributed to the methine (C1) and methylene (C2) of the ethylene–styrene joint sequence. Additionally, the methine (C8) and methylene (C9) of the ethylene–hexene joint sequence are observed at δ = 37.6 ppm and δ = 34.0 ppm, respectively. The peak at δ = 29.4 ppm is assigned to the methylene carbons (C15) from long ethylene–ethylene sequences, while the resonance around δ = 26.7 ppm is attributed to the methylene carbon (C7) from styrene–ethylene–styrene sequences. The resonance derived from hexene–ethylene–hexene sequences (C14) was observed at δ = 22.8 ppm. The absence of resonances assigned to consecutive styrene–styrene, hexene–hexene or styrene–hexene sequences in the aliphatic region of the ^13^C NMR spectra suggests that the hexene and styrene units are discretely dispersed in the polyethylene backbone, which is consistent with the results of the DSC analysis.

### 3.2. Terpolymerization of Ethylene with Hexene and Styrene Derivatives

It is generally believed that introducing amine groups into the polymer backbone can enhance the material’s surface and mechanical properties [30,40]. Inspired by the results of the terpolymerization of ethylene with hexene and styrene, three additional amine-substituted monomers were synthesized in order to obtain materials with functional groups and structural diversity. The terpolymerization of ethylene with hexene and *p*-N, N-dimethylaminostyrene (DMAS) or *p*-N, N-diethylaminostyrene (DEAS) was carried out with varying comonomer feeding amounts, as shown in Table 2 (runs 1–5 for DMAS and runs 6–10 for DEAS). Under the condition of constant ethylene pressure and hexene feeding amounts, when the feeding amount of DMAS (Table 2, runs 1–3) or DEAS (Table 2, runs 6–8) was increased, the polymerization activity increased slightly, and the molecular weight gradually decreased while kept a unimodal molecular weight distribution. A similar trend was observed in the terpolymerization of ethylene with hexene and styrene. However, the terpolymerization activity of ethylene with hexene and DMAS or DEAS was lower than that of ethylene with hexene and styrene under the same conditions, possibly due to the interactions between amine groups and catalysts, which may hinder the coordination insertion of monomers. The compositions of poly(ethylene-hexene-DMAS) and poly(ethylene-hexene-DEAS) were determined by ^1^H NMR spectroscopy (Figures S4 and S7). As the initial concentration of DMAS or DEAS increased (Table 2, runs 1–3 and runs 6–8), the incorporation amounts of these styrene derivatives in the terpolymer increased, while the incorporation amount of hexene slightly decreased. According to the ratios of [FSt]/[Hex] and [FSt]_0_/[Hex]_0_, it is indicated that DMAS or DEAS mainly replaces ethylene rather than hexene during polymerization. When hexene and DMAS or DEAS were in the same feeding amount, the conversion of styrene derivatives was higher than that of hexene (Table S1, runs 7 and 12), indicating that the coordination insertion of DMAS or DEAS was easier than that of hexene. Moreover, under the same conditions, the conversion of the comonomers in the terpolymerization of ethylene, hexene and DMAS or DEAS was lower than that in the terpolymerization of ethylene, hexene and styrene. This may be for the same reason as the findings observed in polymerization activity. When the relative feeding ratio of hexene to styrene derivatives was increased (Table 2, runs 3–5 and runs 8–10), the incorporation of hexene in the terpolymers increased, but the content of styrene derivatives decreased. The ratios of [FSt]/[Hex] and [FSt]_0_/[Hex]_0_ also gradually decreased, indicating that the increase in the amount of hexene fed is much faster than its consumption rate in the terpolymerization of ethylene with hexene and DMAS or DEAS. Ultimately, terpolymers with DMAS incorporation rates of 3.1 wt%–7.2 wt% and DEAS incorporation rates of 4.6 wt%–8.4 wt% were obtained. Only one *T*_m_ was observed from the DSC curves (Figures S6 and S9), and the *T*_m_ and the *X*_c_ of terpolymers all decreased with the increasing incorporation amount of comonomers.

Polymers containing triphenylamine groups are extensively used in polymer-based electronic devices due to their excellent hole-transporting properties and photophysical characteristics [41,42]. Compared to polymers with triphenylamine groups in the main chain, those with triphenylamine groups in the side chain offer more advantages in controlling the polymer architecture and have thus attracted more interest. Consequently, *p*-N, N-diphenylaminostyrene (DPAS) was synthesized and the terpolymerization of ethylene with hexene and DPAS was investigated. Initially, when the ethylene pressure and the hexene feeding amount were kept constant, the polymerization activity increased significantly with the increasing of the DPAS feeding amount. The conversion rate of DPAS was lower than that of hexene at the same feeding amount (Table S1, run 17), which may be due to the larger steric hindrance of DPAS. The ratios of [DPAS]/[Hex] and [DPAS]_0_/[Hex]_0_ were basically constant, indicating that DPAS mainly replaces ethylene rather than hexene during the polymerization process. Furthermore, when the relative feeding amount of hexene to DPAS was increased, the polymerization activity also improved. The ratios of [DPAS]/[Hex] and [DPAS]_0_/[Hex]_0_ also gradually decreased, just like what was observed in the terpolymerization of ethylene with hexene and styrene or other styrene derivatives. However, an increase in the feeding amount of DPAS or the relative feeding ratio of hexene to DPAS led to a slight decrease in the molecular weight of the obtained terpolymers. Ultimately, terpolymers with a moderate molecular weight (7.4–12.7 × 10^4^) and a unimodal molecular weight distribution (PDI = 1.8–2.3) were obtained. ^1^H NMR spectrum results (Figure S10) indicated that the incorporation amounts of hexene and DPAS in the terpolymers increased with the initial concentration of the comonomers, yielding terpolymers with the incorporation amounts of DPAS ranging from 4.1 wt% to 9.7 wt%. The DSC curves (Figure S12) revealed that as the incorporation content of comonomers in polyethylene increased, the *T*_m_ of the terpolymer decreased from 130.0 °C to 127.2 °C, and the *X*_c_ of the terpolymers also decreased.

**Figure 2:**
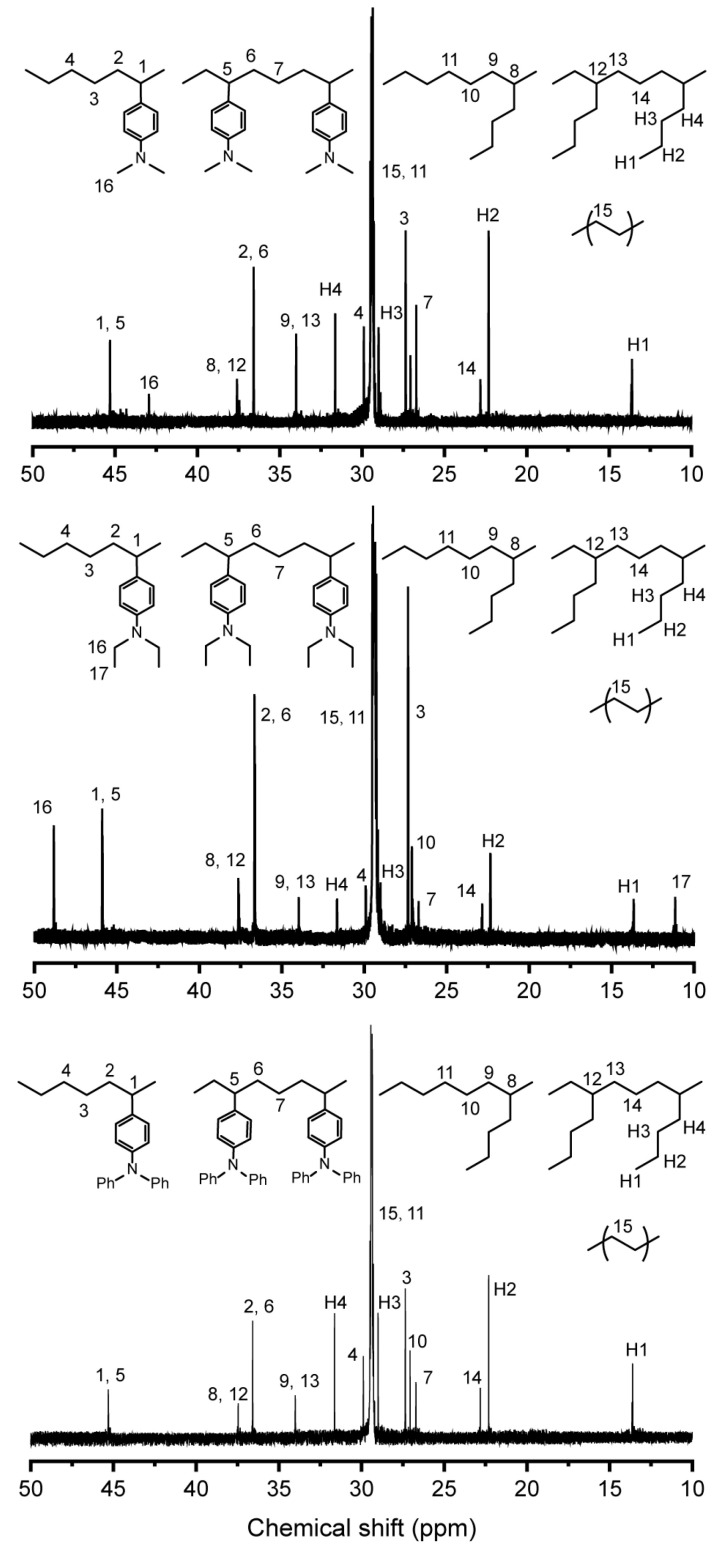
Aliphatic region of ^13^C NMR spectra of poly(ethylene-hexene-styrene derivatives).

The microstructure of the obtained poly(ethylene-hexene-styrene derivatives) was characterized via ^13^C NMR. As shown in Figure 2, the resonances observed in the range of 45.3–46.0 ppm and 36.5–36.7 ppm are attributed to the methine (C1) and methylene (C2) of the ethylene–styrene derivative joint sequence. The resonances at approximately 37.6 ppm and 34.0 ppm correspond to the methine (C8 and C12) and methylene (C9 and C13) groups of the ethylene–hexene joint sequence. The methylene carbon signal observed at δ = 29.4 ppm is assigned to the methylene carbon (C15) from long consecutive ethylene–ethylene sequences. The resonance derived from the styrene derivative–ethylene–styrene derivative consecutive sequence is observed at δ = 26.7 ppm (C7). Additionally, the resonance from the consecutive hexene–ethylene–hexene sequences is observed around 22.8 ppm (C14). No resonances of consecutive styrene derivatives–styrene derivatives sequences or consecutive hexene–hexene sequences are found in the ^13^C NMR spectra, indicating that hexene and styrene derivatives units are discretely dispersed in the polyethylene backbone.

### 3.3. Mechanical Properties of Terpolymers

**Figure 3:**
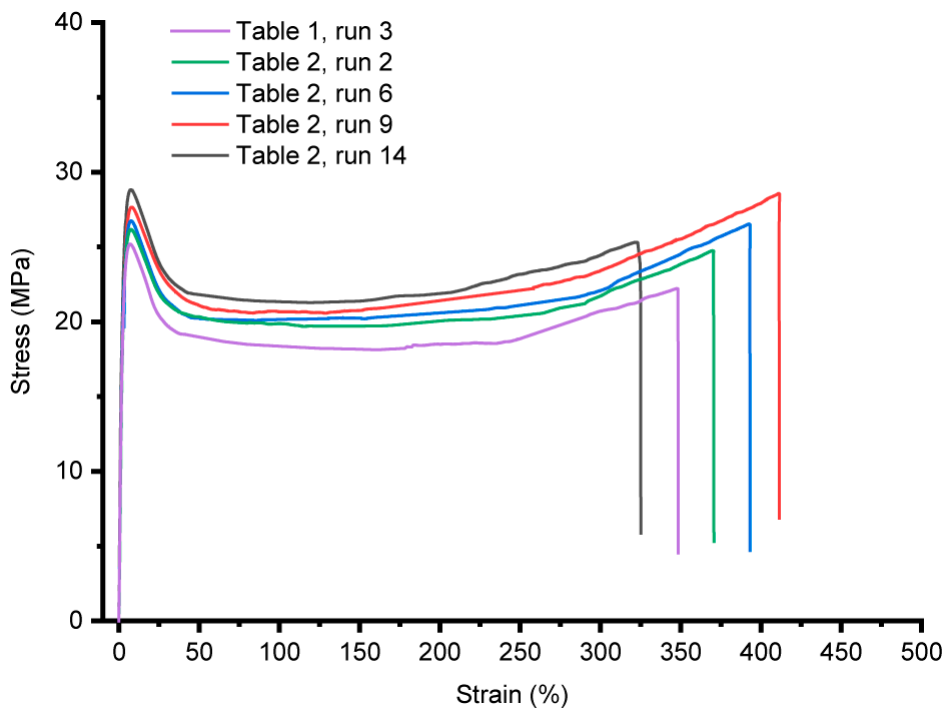
Stress–strain curves of terpolymers.

The material properties and potential applications of the amine-functionalized polyolefin materials have been investigated. As shown in Figure 3, tests on four samples (Table 1, run 3; Table 2, runs 2, 6, 14) with comparable compositions revealed that terpolymers containing styrene derivatives demonstrated enhanced tensile strength compared to the terpolymer containing styrene. Among these, the terpolymer containing DPAS exhibited the highest tensile strength but the shortest elongation at break. In contrast, the terpolymers containing DEAS displayed the longest elongation at break. In addition, an increase in the incorporation amount of styrene derivatives (Table 2, runs 6 and 9) led to improvements in both the tensile strength and elongation at break of the terpolymers. These results suggest that the introduction of styrene derivatives into polyethylene can improve the mechanical properties of the terpolymer, which is probably due to the hydrogen bonding effect of the amine groups. Moreover, the benzene rings increase the rigidity of the polymer and serve as physical cross-linking points [43,44].

### 3.4. Surface Property of Terpolymers

**Figure 4:**
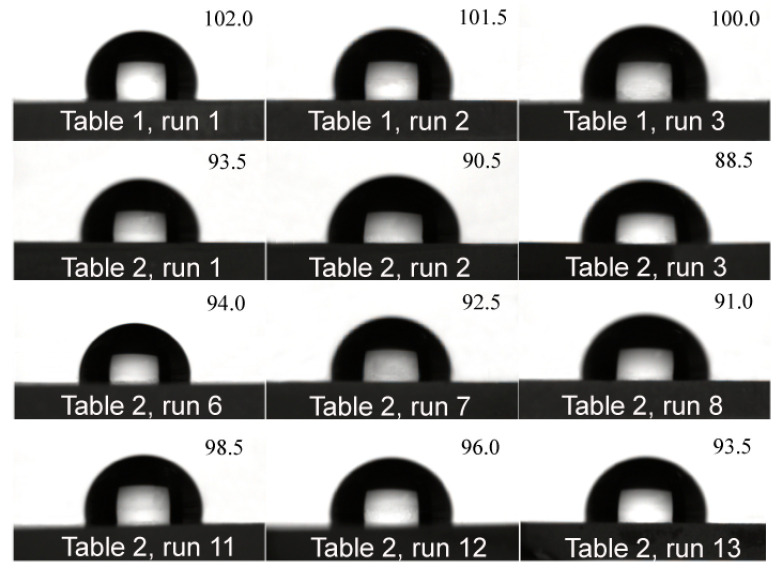
Water contact angles of terpolymers.

It is expected that the introduction of polar groups into polyethylene can alter its surface properties [42]. The water contact angles (WCA) of identical terpolymer samples were measured using thin films coated on glass slides (Figure 4). Obviously, the WCA of the obtained terpolymers decreased significantly with the increase in styrene derivative content. Particularly, the terpolymer containing DMAS exhibited the most substantial reduction in WCA.

## 4. Conclusions

The terpolymerization of ethylene with hexene and styrene derivatives was successfully achieved for the first time by using a half-sandwich scandium catalyst. The terpolymerization of ethylene with hexene and styrene derivatives proceeded in a controlled fashion, yielding a novel family of terpolymers with adjustable components. By simply altering the feeding amounts of hexene and styrene derivatives, terpolymers of ethylene, hexene and styrene derivatives with a moderate molecular weight (7.4–12.7 × 10^4^) and a unimodal molecular weight distribution (PDI = 1.8–2.7) were obtained. The Tm decreased with the increasing incorporation amounts of comonomers, indicating that they were distributed along the polyethylene backbone, rather than forming a mixture of homopolymers or copolymers. ^13^C NMR spectra revealed that the hexene and styrene derivatives were dispersed in the polyethylene in an isolated manner. Tensile tests demonstrated that amine-functionalized polyethylenes possessed more beneficial mechanical properties. The water contact angle of terpolymers indicated that the surface properties of all functional terpolymers were significantly improved. This work presents an efficient and convenient route for the synthesis of functional polyethylenes via direct terpolymerization under mild conditions, which may have significant potential for enhancing polyethylene’s properties. More studies on the performances of these well-defined polyethylenes are in progress.

## Figures and Tables

**Table 1:** Terpolymerization of ethylene with hexene and styrene.

Run ^(a)^	FSt	Hex (mmoL)	FSt (mmol)	Act. ^(b)^	*f*_E_ ^(c)^ (mol%)	*f*_Hex_ ^(c)^ (mol%)	*f*_FSt_ ^(c)^ (mol%)	*M*_n_ ^(d)^ [×10^4^ Da]	*M*_w_/*M*_n_ ^(d)^	*T*_m_ ^(e)^ (^o^C)	*X*_c_ ^(e)^ (%)
1	St	20	10	3684	98.2	1.2	0.6	12.9	1.8	127.7	51.4
2	St	20	20	3810	97.8	1.1	1.1	11.3	2.1	123.6	49.1
3	St	20	30	4166	97.5	1.0	1.5	10.7	1.7	123.0	46.8
4	St	30	20	4550	97.7	1.2	1.1	10.1	2.4	123.4	48.6
5	St	40	10	4780	97.9	1.5	0.6	9.7	1.7	126.9	51.1

^(a)^ Polymerization conditions: Catalysts, 40 μmol; [Ph_3_C][B(C_6_F_5_)_4_], 40 μmol; Ethylene, 0.5 Mpa; Time, 15 min; Toluene, 600 mL, 25 °C; ^(b)^ Given in kg of polymer per (mol_Sc_ h); ^(c)^ Measured by ^1^H NMR spectroscopy. ^(d)^ Determined by GPC in 1,2,4-trichlorobenzene at 160 °C against polystyrene standard; ^(e)^ Determined by DSC.

**Table 2:** Terpolymerization of ethylene with hexene and styrene derivatives.

Run ^(a)^	FSt	Hex (mmoL)	FSt (mmol)	Act. ^(b)^	*f*_E_ ^(c)^ mol%	*f*_Hex_ ^(c)^ mol%	*f*_FSt_ ^(c)^ mol%	*M*_n_ ^(d)^ [×10^4^ Da]	*M*_w_/*M*_n_ ^(d)^	*T*_m_^(e)^	*X*_c_ ^(e)^ (%)
1	DMAS	20	10	1720	98.0	1.3	0.7	12.4	2.5	134.2	43.8
2	DMAS	20	20	2080	97.9	1.0	1.1	11.6	2.1	132.4	38.4
3	DMAS	20	30	2130	97.5	1.0	1.5	10.2	2.3	128.5	35.1
4	DMAS	30	20	2360	97.6	1.3	1.1	10.1	1.9	130.5	35.7
5	DMAS	40	10	2550	97.7	1.5	0.8	8.5	2.7	130.6	35.9
6	DEAS	20	10	1404	98.0	1.1	0.9	11.9	1.9	135.5	36.5
7	DEAS	20	20	1609	97.8	0.9	1.3	11.2	2.6	133.9	35.7
8	DEAS	20	30	1845	97.7	0.8	1.5	10.6	2.1	131.6	35.5
9	DEAS	30	20	2057	97.8	1.1	1.1	9.8	2.6	134.9	36.0
10	DEAS	40	10	2280	98.0	1.2	0.8	8.2	2.4	137.0	36.7
11	DPAS	20	10	5020	98.6	1.0	0.4	12.7	1.8	129.6	48.6
12	DPAS	20	20	5150	98.5	0.8	0.7	12.2	2.3	129.2	46.3
13	DPAS	20	30	5380	98.3	0.7	0.9	10.3	2.1	127.2	43.1
14	DPAS	30	20	5510	98.4	1.0	0.6	8.9	1.8	129.4	44.2
15	DPAS	40	10	5730	98.4	1.3	0.3	7.4	1.9	130.0	44.5

^(a)^ Polymerization condition: Catalysts, 40 μmol; [Ph_3_C][B(C_6_F_5_)_4_], 40 μmol; Ethylene, 0.5 Mpa; Time, 15 min; Toluene, 600 mL, 25 °C; ^(b)^ Given in kg of polymer per (mol_Sc_ h); ^(c)^ Measured by ^1^H NMR spectroscopy. ^(d)^ Determined by GPC in 1,2,4-trichlorobenzene at 160 °C against polystyrene standard; ^(e)^ Determined by DSC.

## Data Availability

Date are contained within this article.

## Supplementary Materials

The following supporting information can be downloaded at https://www.mdpi.com/article/10.3390/polym16162290/s1, Table S1: Conversion of comonomers in terpolymerization; Table S2: Chemical shift in ^1^H NMR of terpolymer samples; Table S3: Mechanical properties of terpolymer samples; Table S4: Water contact angles of terpolymer samples; Figure S1: ^1^H NMR spectra of poly(ethylene-hexene-styrene); Figure S2: GPC curves of poly(ethylene-hexene-styrene); Figure S3: DSC curves of poly(ethylene-hexene-styrene); Figure S4: ^1^H NMR spectra of poly(ethylene-hexene-DMAS); Figure S5: GPC curves of poly(ethylene-hexene-DMAS); Figure S6: DSC curves of poly(ethylene-hexene-DMAS); Figure S7: ^1^H NMR spectra of poly(ethylene-hexene-DEAS); Figure S8: GPC curves of poly(ethylene-hexene-DEAS); Figure S9: DSC curves of poly(ethylene-hexene-DEAS); Figure S10: ^1^H NMR spectra of poly(ethylene-hexene-DPAS); Figure S11: GPC curves of poly(ethylene-hexene-DPAS); Figure S12: DSC curves of poly(ethylene-hexene-DPAS).
