# Supplementary material for: Terpolymerization of Ethylene with Hexene and Styrene Derivatives by Half-Sandwich Scandium Catalyst
Source: Polymers (Basel). 2024 Aug 14;16(16):2290. doi: 10.3390/polym16162290 (PMC11359482; doi:10.3390/polym16162290)
Supplement: Supplementary file 1 [file polymers-16-02290-s001.zip › polymers-3143939-supplementary.pdf]

Supporting information

Terpolymerization of Ethylene with Hexene and Styrene Derivatives by Half-Sandwich Scandium Catalyst

Xiaochun Mu [1,2], Xuefei Leng [3,*], Chuanchuan Liu [2], Qiang Yao [1] and Yang Li [3,*]

1 Key Laboratory of Bio-Based Polymeric Materials Technology and Application of Zhejiang Province, Ningbo Institute of Materials Technology and Engineering, Chinese Academy of Sciences, Ningbo 315201, China; muxcdlut@163.com (X.M.); yaoqiang@nimte.ac.cn (Q.Y.)

2 SINOPEC Ningbo New Materials Research Institute Company Limited, Ningbo 315201, China; liuchch697.zhlh@sinopec.com

3 State Key Laboratory of Fine Chemicals, Department of Polymer Science and Engineering, School of Chemical Engineering, Dalian University of Technology, Dalian 116024, China

* Correspondence: lengxuefei@dlut.edu.cn (X.L.); liyang@dlut.edu.cn (Y.L.)

Table S1. Terpolymerization of ethylene with hexene and styrene or styrene derivatives

	FSt	Hex (mmoL)	FSt (mmol)	Conv. of Hex [b] (%)	Conv. of FSt [b] (%)
1	St	20	10	73.4	78.4
2	St	20	20	68.5	74.3
3	St	20	30	67.3	72.9
4	St	30	20	63.7	83.2
5	St	40	10	61.6	94.7
6	DMAS	20	10	38.1	40.2
7	DMAS	20	20	36.2	38.3
8	DMAS	20	30	34.1	34.7
9	DMAS	30	20	33.8	43.6
10	DMAS	40	10	32.4	65.1
11	DEAS	20	10	25.3	43.1
12	DEAS	20	20	24.9	33.9
13	DEAS	20	30	24.7	29.5
14	DEAS	30	20	24.0	37.8
15	DEAS	40	10	23.1	59.6
16	DPAS	20	10	82.2	76.2
17	DPAS	20	20	71.0	57.3
18	DPAS	20	30	64.9	53.9
19	DPAS	30	20	63.6	55.0
20	DPAS	40	10	61.7	62.1

[a] Polymerization condition: Catalysts, 40 μmol, $[Ph_3C][B(C_6F_5)_4]$, 40 μmol, Ethylene, 0.5Mpa; Time, 15min, Toluene, 600 mL, 25 °C; [b] Calculated according to incorporation amount and feeding amount.

The contents of comonomers in the terpolymer were calculated according to ^{1}H NMR spectra. Taking ethylene-hexene-styrene terpolymer as an example, the ethylene (E), hexene (H) and styrene (St) contents were calculated according to the following formulas:

$$\text{H mol\%} = 20I_3/(3I_1+15I_2+5I_3)\times 100$$

$$\text{St mol\%} = 12I_1/(3I_1+15I_2+5I_3)\times 100$$

$$\text{E mol\%} = 100 - \text{H mol\%} - \text{St mol\%}$$

where I_1 is the integration of the resonances from 7.32 to 6.85 ppm (unsaturated protons of the aromatic unit), I_2 is the integration of the resonances from 2.57 to 0.98 ppm (three protons of the ethylene unit, three protons of the hexene unit, three protons of the styrene), and I_3 is the integration of the resonances from 0.97 to 0.88 ppm (three methyl protons of the hexene unit).

Table S2. Chemical shift in ^{1}H NMR of terpolymer samples

Peak	Chemical shift ^{1}H	Type	Assignment in previous report
1	0.90-0.98	CH_3	0.75-1.10
2	1.0-1.80	CH_2	1.10-1.77
3	2.44-2.57	CH_2	2.25-2.65
4	2.77-3.05	N-CH_3	2.6-3.0
5	3.33-3.50	N-CH_2	3.0-3.50
6	6.45-7.47	Ar-H	6.90-7.50

Table S3. Mechanical properties of terpolymer samples

Samples	f_e mol%	f_{hex} mol%	F_{FSt} mol%	Tensile strength MPa	Elongation at Break %
Table 1, run 3	97.5	1.0	1.5	25.6±0.7	348±15
Table 2, run 2	97.9	1.0	1.1	26.5±0.9	370±18
Table 2, run 6	98.0	1.1	0.9	27.6±1.2	392±20
Table 2, run 9	97.8	1.1	1.1	28.0±1.0	418±20
Table 2, run 14	98.4	1.0	0.6	29.2±1.2	325±18

Table S4. Water contact angles of terpolymer samples

Samples	f_e mol%	f_{hex} mol%	F_{FSt} mol%	Water contact angles
Table 1, run 1	98.2	1.2	0.6	102.0
Table 1, run 2	97.8	1.1	1.1	101.5
Table 1, run 3	97.5	1.0	1.5	100.0
Table 2, run 1	98.0	1.3	0.7	93.5
Table 2, run 2	97.9	1.0	1.1	90.5
Table 2, run 3	97.5	1.0	1.5	88.5
Table 2, run 6	98.0	1.1	0.9	94.0
Table 2, run 7	97.8	0.9	1.3	92.5
Table 2, run 8	97.7	0.8	1.5	91.0
Table 2, run 11	98.6	1.0	0.4	98.5
Table 2, run 12	98.5	0.8	0.7	96.0
Table 2, run 13	98.3	0.7	0.9	93.5

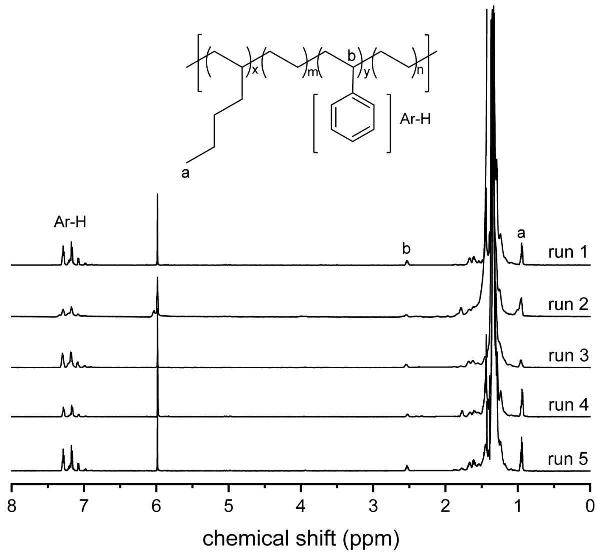

Figure S1 ^{1}H NMR spectra of poly(ethylene-hexene-styrene).

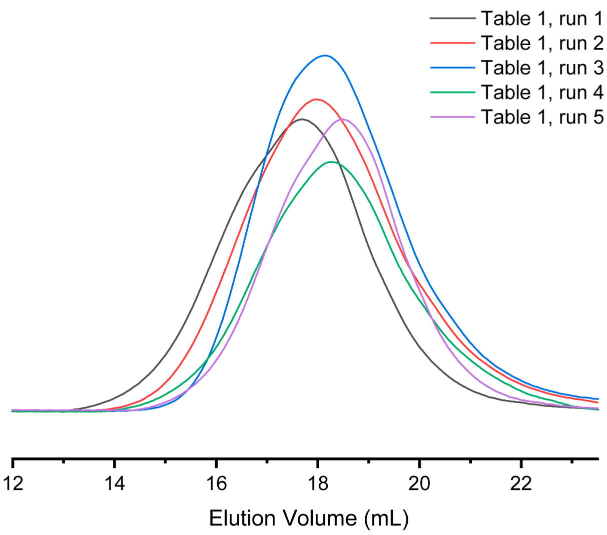

Figure S2 GPC curves of poly(ethylene-hexene-styrene).

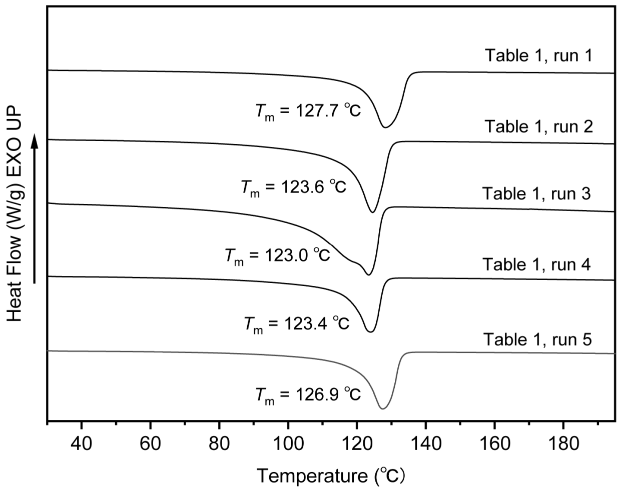

Figure S3 DSC curves of poly(ethylene-hexene-styrene).

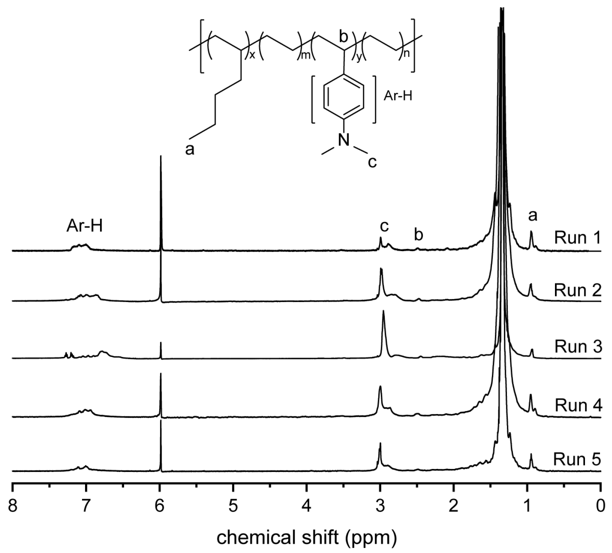

Figure S4 ^{1}H NMR spectra of poly(ethylene-hexene-DMAS).

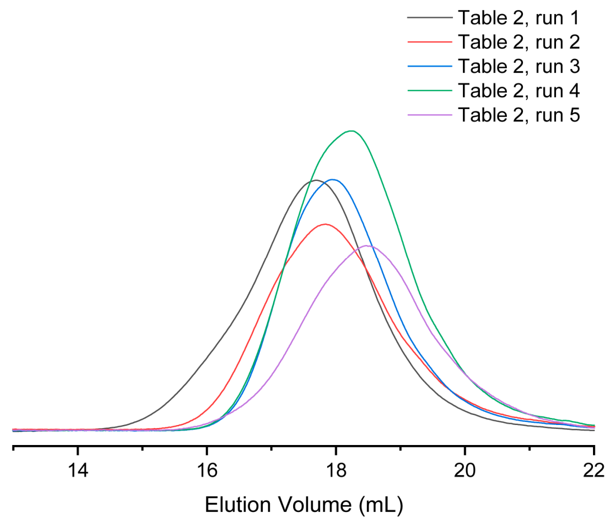

Figure S5 GPC curves of poly(ethylene-hexene-DMAS).

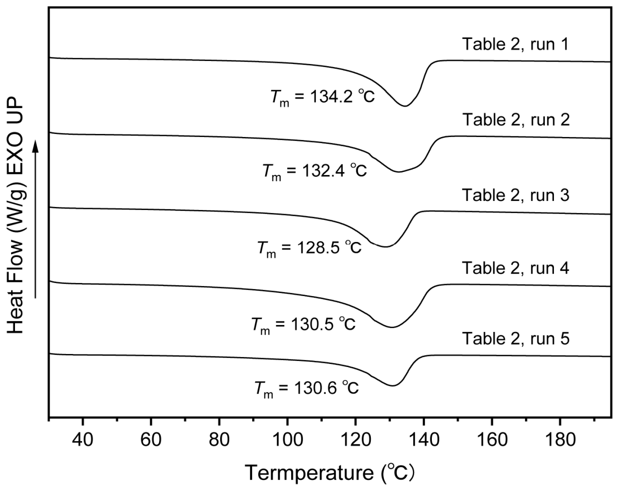

Figure S6 DSC curves of poly(ethylene-hexene-DMAS).

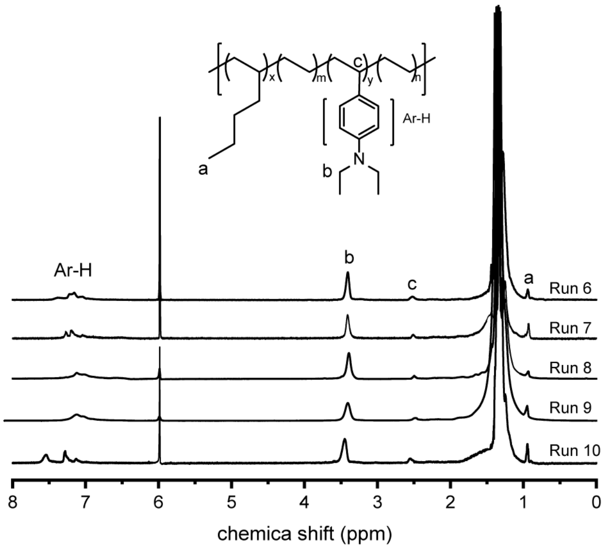

Figure S7 ^{1}H NMR spectra of poly(ethylene-hexene-DEAS).

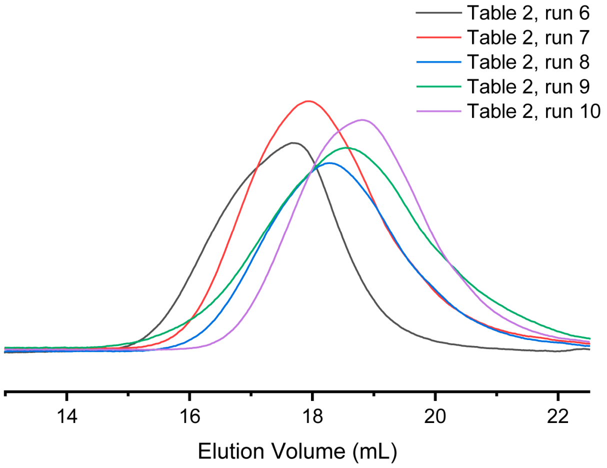

Figure S8 GPC curves of poly(ethylene-hexene-DEAS).

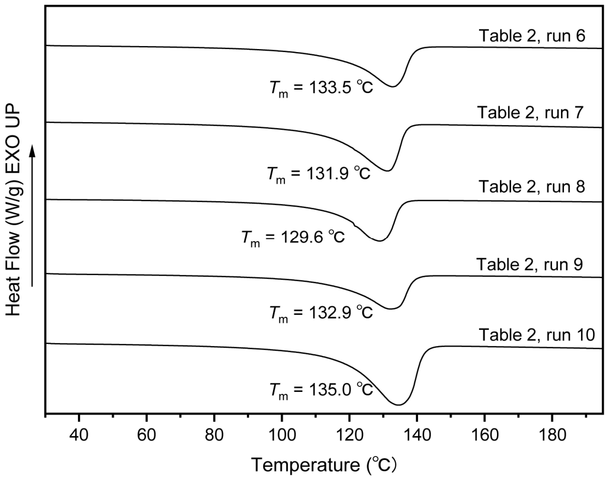

Figure S9 DSC curves of poly(ethylene-hexene-DEAS).

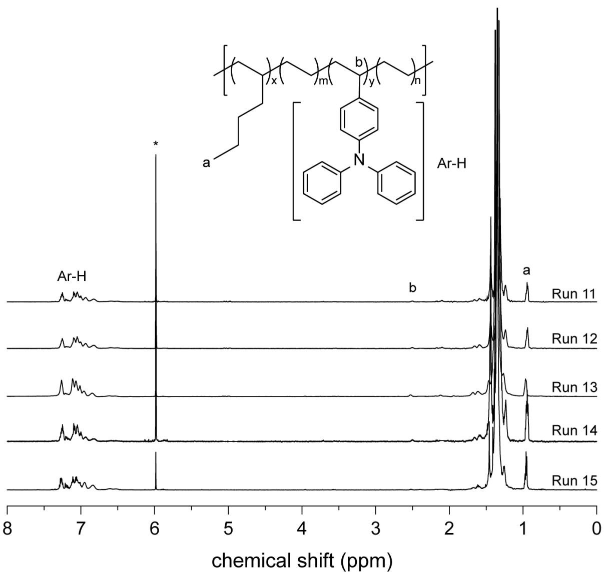

Figure S10 ^{1}H NMR spectra of poly(ethylene-hexene-DPAS).

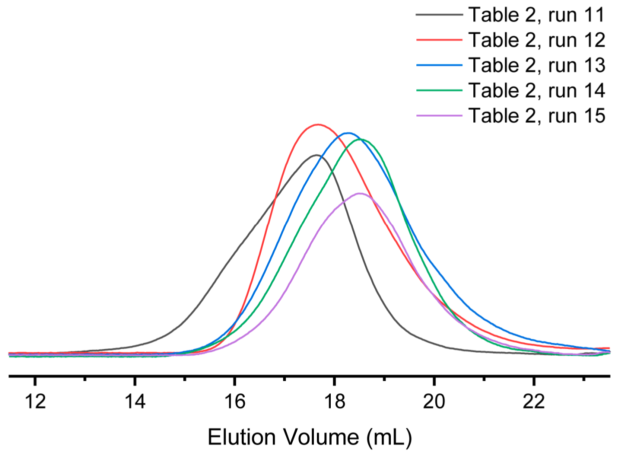

Figure S11 GPC curves of poly(ethylene-hexene-DPAS).

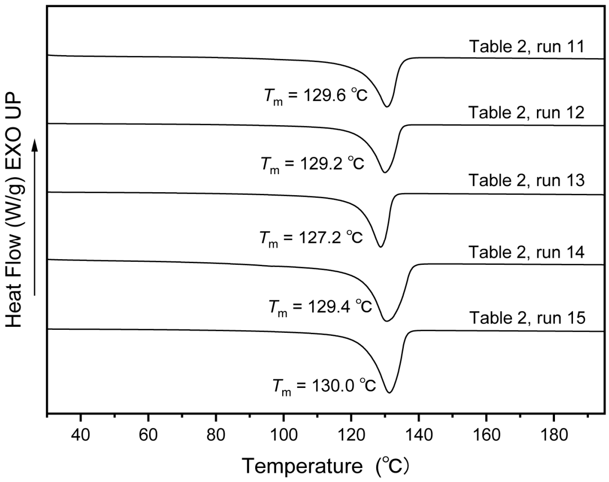

Figure S12 DSC curves of poly(ethylene-hexene-DPAS).
